# The psychosis spectrum and underlying factors in female youth with eating disorders: a cross-sectional observational experience sampling methodology study

**DOI:** 10.3389/fpsyt.2025.1715651

**Published:** 2025-12-26

**Authors:** A. J. M. Roedelof, C. J. P. Simons, S. Mulkens, M. Marcelis

**Affiliations:** 1 Institute for Mental Health care Eindhoven (GGzE), Eindhoven, Netherlands; 2 Department of Psychiatry and Neuropsychology, Mental Health and Neuroscience Research Institute, Maastricht University, Maastricht, Netherlands; 3 Department of Clinical Psychological Science, Maastricht University, Maastricht, Netherlands; 4 Department of Psychiatry and Psychology, Maastricht University Medical Center (MUMC+), Maastricht, Netherlands

**Keywords:** eating disorders, ecological momentary assessment, psychosis spectrum, psychotic experiences, transdiagnostics

## Abstract

**Objective:**

Recent reviews indicated that the psychosis spectrum co-occurs with disorders on the eating disorder (ED) spectrum, which might elucidate the low recovery rates. Consequently, the ED field is encouraged to better understand the interplay between these two spectra. This study examined whether female youth with EDs experience higher levels of psychotic phenomena than controls, including associations between different levels of (subclinical) psychosis and the factors negative affect (NA), self-esteem, stress, and ED severity.

**Method:**

A cross-sectional study using experience sampling methodology (ESM) for 10 days involved 50 young female individuals with EDs and 50 controls. Subclinical psychotic experiences (PE), psychotic symptoms, and disorders were assessed using ESM, Community Assessment of Psychic Experiences, Positive and Negative Syndrome Scale, and Mini-International Neuropsychiatric Interview. ED severity was assessed with the Eating Disorder Examination–Questionnaire. Mixed effects, logistic, and linear regression models were used for the analyses.

**Results:**

The ED group had significantly more momentary PE (*B* = 1.57, *p* <.001), lifetime PE (*B* = 0.50, *p* <.001), psychotic symptoms (*B* = 17.21, *p* <.001) and lifetime psychotic disorders (*B* = 2.83, *p* <.001) than controls. In the ED group, momentary PE were associated with momentary NA, self-esteem, stress, and EDE-Q; lifetime PE were associated with momentary NA, stress, and EDE-Q; and psychotic symptoms were associated with momentary NA.

**Discussion:**

All levels of psychosis were common in female youth with an ED, and these (subclinical) psychotic phenomena were associated with transdiagnostic factors such as momentary NA, self-esteem, and stress and/or ED severity. The findings have relevance for clinical practice and inform future transdiagnostic studies on the mechanism and treatment of individuals with EDs and (comorbid) psychosis.

## Introduction

Eating disorders (EDs) remain challenging to treat. This is particularly so for individuals with anorexia nervosa restrictive type (AN-r), anorexia nervosa binge/purging type (AN-b/p), bulimia nervosa (BN), and other specified feeding or eating disorders (OSFED) that closely resemble AN or BN. They are severely concerned about eating, shape, and weight and endorse intense fears of gaining weight. Furthermore, multiple underlying transdiagnostic factors such as limited affect and stress tolerance, low self-esteem, and negative body and self-image may influence the emergence and maintenance of food-related behaviors (1, 2).

A recent systematic review and meta-analysis (3) reported that 46% of the individuals with an ED recover fully, almost 25% recover partially, and 25% develop into a chronic course. Mortality rates are high (0.4%) (3) due to the somatic consequences of the ED and due to suicide (4). Therefore, it is crucial to shift focus toward understanding the broader symptom dynamics within their context through an interdisciplinary approach, as it could help personalize treatment and prevent worsening of the condition (5).

Emerging evidence suggests the co-occurrence of psychosis spectrum features across the ED spectrum (6–8). A meta-analysis (6) reported an 8% (95% CI: 2%–19%) comorbidity rate across clinical populations with ED. For clarity, we refer in this study to general psychotic features, rather than to ED-specific psychotic-like phenomena, such as the hallucinatory anorexic voice or delusional distortions in body image. Psychotic features refer to aberrant perceptions, emotions, thoughts, and beliefs in individuals, varying in intensity and frequency from psychotic experiences (PE) to psychotic disorder (i.e., the psychosis continuum) (9). PE may represent one of the early, transdiagnostic dimensions of psychopathology (10, 11), with prevalence estimates of 8%–33% for PE, 4%–17% for psychotic symptoms, and 1.7%–3% for psychotic disorders in the general population (6, 9, 12–16). PE can be a precursor to psychotic disorders, with approximately 20% of individuals with PE developing persistent PE and 7% transitioning to a psychotic disorder, with an annual transition rate of 0.5%–1% (17). PE also predicts greater severity of comorbid illness and predicts a poorer treatment response (17). Among people at high clinical risk for psychosis, 25% develop psychosis within 3 years (18). Transdiagnostic factors such as negative affect (NA) (10, 19, 20), stress (8, 21), and low self-esteem (22–25) have been found to impact the psychosis spectrum phenomena.

In the current study, we adopted an interdisciplinary approach and investigated the expression of general psychotic features in young girls and women (15–25 years) around the start of their ED treatment as usual, i.e., family-based treatment (26) for adolescents and cognitive behavior therapy—enhanced (CBT-e) (27) for adult women. ED-specific psychotic-like phenomena, such as the hallucinatory anorexic voice or delusional distortions in body image, are not part of this article. The aim of the present study was to compare the expression of general psychotic features in female youth with an ED with a non-ED control group and to examine associations between underlying factors and the psychotic features in individuals with an ED. In doing this, the entire psychosis spectrum was included, and we used validated cross-sectional measurements along with ecologically sensitive, experience sampling method (ESM), data. Our primary objective was (i) to examine the occurrence of the entire psychosis continuum (with daily life and current/lifetime measures) in individuals with EDs compared to matched women without an ED. Our second objective was (ii) to investigate in the ED group whether psychosis spectrum phenomena were associated with (a) assumed underlying factors of the ED NA, self-esteem, and stress) and (b) the severity of the ED (measured with the Eating Disorder Examination–Questionnaire (EDE-Q)). Our last objective was (iii) to examine whether ESM can be used validly to investigate PE and underlying factors in daily life in an ED group.

To examine the occurrence of the psychosis continuum in ED and underlying factors, we applied ESM. ESM is a structured diary technique that can provide real-life contextual data on mood, thoughts, and environmental factors (28). This allowed us to monitor momentary PE in relation to daily-life risk factors. Although ESM has been used before to assess momentary PE in other psychiatric conditions (23, 29–31), its application in an ED group is novel. We focused on young female individuals aged 15–25 years around the start of their ED treatment as this period marks the peak onset of both EDs and first psychotic episodes (32), and the treatment effect is most limited.

## Method

### Sample

The study, conducted between April 2019 and December 2021 in Eindhoven, The Netherlands, recruited individuals with an ED between the first ambulatory contact (intake) for the ED and 3 months after starting ED treatment as usual in a large mental health organization. We did not collect information on how many of the participants were already receiving ED treatment after the first contact and for how long (which could be up to a maximum of 3 months). The participants, aged 15–25 years, were Dutch-speaking, had an (estimated) IQ above 70, and received a DSM-5 ED diagnosis during the intake procedure. The exclusion criteria were acute psychosis, high suicidal risk, and specific somatic disorders. Of the 54 participants, two dropped out, and two were excluded for not meeting the criteria for a DSM-5 ED diagnosis. The control group was recruited in the same period through schools, social media, and flyers and was matched for age and education. We were unable to group-match on education, but we corrected *a priori* for education as accounted for in the statistical analyses. The exclusion criteria were history of ED, family history of ED, specific somatic disorders, and current mental health treatment. One control was excluded due to suspected ED. Both groups were screened using the “Sick, Control, One stone, Fat, Food” questionnaire (SCOFF) (33) and EDE-Q (34). There were 50 women in the ED group (mean age, 17.78) and 50 matched women in the control group (mean age, 18.72). In the ED group, 24% had AN-b/p, 62% had AN-r, 8% had BN, and 6% had OSFED (see **Table 1**). The presence of other mental disorders, including psychotic disorders (current and lifetime), was screened using the Mini-International Neuropsychiatric Interview (MINI) (35).

**Table 1:** Sociodemographic and clinical characteristics of the research sample.

	Female individuals with an eating disorder (*N* = 50)	Control group (*N* = 50)	Group comparisons *p*
Demographic variables			
Age, mean (SD)	17.78 (2.68)	18.72 (2.65)	.081
Dutch, *n* (%)	46 (92)	42 (84)	.357
Education: highest degree, mean (SD*)*	4.84 (0.96)	5.26 (1.12)	.047*
No education (0)	0	0	
Primary school (1)	0	0	
Secondary school lower vocational (2)	0	1 (2%)	
Secondary school (3)	7 (14%)	3 (6%)	
Lower vocational education (4)	4 (8%)	5 (10%)	
High school (5)	31 (62%)	20 (40%)	
Higher vocational education (6)	6 (12%)	15 (30%)	
University (7)	2 (4%)	6 (12%)	
Clinical variables			
BMI, mean (SD)	19.74 (2.68)	22.70 (2.92)	<.001***
EDE-Q global, mean (SD)	4.33 (0.89)	0.65 (0.57)	<.001***
DSM-5 classification: eating disorder, *n* (%)			
Anorexia nervosa binge/purging type	12 (24%)	–	–
Anorexia nervosa restrictive type	31 (62%)	–	–
Bulimia nervosa	4 (8%)	–	–
Other specified feeding or eating disorder	3 (6%)	–	–
Multi-morbidity (current, MINI), mean (SD)	1.98 (1.81)	0.44 (0.86)	<.001***

BMI, body mass index; EDE-Q, Eating Disorder Examination—Questionnaire; MINI, Mini-International Neuropsychiatric Interview.

*p <.05; ***p <.001.

### Procedure

The questionnaires, i.e., screening instruments and interviews were administered in person during a session before and after the ESM period by trained research assistants. Both groups underwent the same procedure, including the screening for presence of an ED with the SCOFF and EDE-Q as well as screening with the MINI (to exclude high suicidal risk or acute psychosis). Clinical judgment was used in case of doubt. EDE-Q was measured as an ED severity indicator. The participants used the “PsyMate™ app” (www.psymate.eu) on their mobile phone or Ipod for the ESM, which prompted to report mood, thoughts, behavior, context, activities, and stress directly after the device emitted a beep (=observation moment) 10 times daily for 10 days. The participants could seek assistance throughout the study, and after the 10-day period, a debriefing session followed. The ED group was offered the possibility of personalized ESM data evaluation for treatment goals.

### Measures of psychosis spectrum phenomena

To examine psychosis spectrum phenomena in the ED group and control group, several measures were used.

#### ESM—momentary PE

Momentary PE were assessed using four self-reported ESM items (36–38): “I feel suspicious”, “I can’t get these thoughts out of my head”, “I hear a voice”, and “I feel unreal”. These experiences were rated on a seven-point Likert scale (1 = not at all, 7 = very much). The personal mean score was determined per person by calculating the average of these items of all completed beeps. Subsequently, we characterized the person mean scores between 1 and 2 as “very mild”, between 3 and 5 as “moderate”, and between 6 and 7 as “very much”.

#### CAPE—lifetime PE

The Community Assessment of Psychic Experiences (CAPE) (39) was used to validly assess lifetime PE after the ESM period. This 42-item self-report questionnaire measures the frequency and distress of positive, negative, and depressive dimensions on a four-point scale (40). A score ≥1 on the CAPE positive frequency scale indicated lifetime PE.

#### PANSS—psychotic symptoms

The Positive and Negative Syndrome Scale (PANSS) (41) assessed psychotic symptoms after the ESM period. This valid semi-structured interview rates the severity of positive, negative, and general symptoms on a seven-point scale, with scores of 2 or 3 indicating subclinical symptoms. A score ≥4 on the positive scale indicated clinically relevant psychotic symptoms.

#### MINI—psychotic disorder

The MINI interview was used to screen validly for the presence of current and lifetime psychotic disorders by scoring 0 (not present) or 1 (present) (35).

### Measures of daily life factors and severity of the ED

#### ESM—momentary NA, self-esteem, and stress

NA was assessed with four items (“I feel insecure”, “I feel anxious”, “I feel down”, and “I feel guilty”), self-esteem with one item (“I like myself”), and stress with three types: activity-related (“I would rather do something else”, “This is difficult for me”, and “I can do this well” (reverse-coded)), social (“I like this company” (reverse-coded), and “I would rather be alone”), and event-related stress (“How pleasant/unpleasant was the event since the last beep”). All items were rated on a scale from 1 (not at all) to 7 (very), except for event-related stress. Event-related stress was assessed by reporting how pleasant/unpleasant the event since the last beep was on a bipolar scale from -3 (very unpleasant) to 0 (neutral) to +3 (very pleasant). Positive events (scores 1, 2, and 3) were recoded to zero, and negative scores were reverse-coded. For all measures, higher scores indicated higher levels of NA, self-esteem, or stress.

#### EDE-Q—severity of the ED

The EDE-Q (34) is a 36-item self-report questionnaire that assessed ED severity over the past 28 days. Its ordinal items are scored on seven-point scales ranging from 0 (no days, not at all, or never) to 6 (every day, markedly, or always). We used the global score (mean of 22 ordinal items assessing restraint, eating concerns, weight concerns, and shape concerns) of the Dutch version. The psychometric qualities are satisfactory to good (42). Higher global scores reflect more severe ED symptoms. A global score ≥3.36 indicated severe pathology (43).

### Statistical analyses

All analyses were performed in R version 4.2.0 (R Studio Desktop 1.4.1106).

#### Comparing the occurrence of psychosis spectrum phenomena between the ED group and the control group

Regression analyses were executed on the ESM, CAPE, PANSS, and MINI scores to compare momentary (daily life), current (past week), and lifetime psychosis spectrum phenomena in individuals with EDs and controls. The assumptions for the regression models were verified, mainly through visual inspection of normality and distribution of residuals. Group was the independent variable, and ESM, CAPE, PANSS, and MINI scores were dependent variables, corrected for age and education (due to psychosis onset up to 25 years and reduced education in psychotic disorders (44)). A two-level mixed-effects regression model was used to compare momentary PE measures between ED group and control group, with random intercepts at the subject level; models were fitted using restricted maximum likelihood estimation (REML). Fixed effects were tested two-sided via Wald-type tests (*α* = .05). ESM data were excluded from the analyses if the participants had less than 33% valid reports (45). Assuming that missing data were completely at random, mixed-effects models estimated via maximum likelihood were used to account for the unequal number of measurements per participant. Based on each fitted model, we computed the slopes of momentary PE with corresponding 95% confidence intervals (CIs). Linear regression analyses were conducted for CAPE (frequency and distress dimensions) and PANSS (total score and three dimensions) and logistic regression analyses for MINI (current and lifetime psychotic disorder).

#### Association between daily life factors (NA, self-esteem, and stress) and psychosis spectrum phenomena within the ED group

In the ED group, the association between daily life experiences (NA, self-esteem, and stress) and psychosis spectrum phenomena was tested. Five two-level mixed-effects regression models were fitted for momentary NA, self-esteem, and three types of stress (social, event, and activity stress) as predictors of momentary PE. Random intercepts and slopes were added, with models fitted using REML. Fixed effects were tested via Wald-type tests (*α* = .05). Slopes were computed with 95% CIs for momentary PE. Linear regression models examined associations between these daily life experiences and CAPE and PANSS scores. Firth’s penalized maximum likelihood logistic regression models were fitted for MINI outcomes. Age and education were covariates, and the Benjamini–Hochberg correction was applied to control for multiple testing.

#### Association between the severity of the ED and psychosis spectrum phenomena in the ED group

Mixed-effects regression analyses were used with EDE-Q global score as predictor for momentary PE (ESM). Linear and logistic regression analyses were conducted for EDE-Q as predictor of lifetime PE (CAPE), current psychotic symptoms (PANSS), and psychotic disorders—current or lifetime (MINI). Age and education were covariates, with the Benjamini–Hochberg correction applied for multiple testing.

#### Substantiation of using ESM for measuring PE

To assess convergent validity, associations between momentary PE (ESM) and lifetime PE (CAPE) and psychotic symptoms (PANSS) were examined using mixed-effects regression analyses, with age and education as covariates.

## Results

### Sample

Data of 50 individuals in the ED group and control group were used for data analyses. On average, the ED group filled in 62 beeps (62%; SD = 13.4) and the control group 68 beeps (68%; SD = 13.7). The sample characteristics are summarized in **Table 1**.

### Momentary, lifetime, and current occurrence of psychosis spectrum phenomena in the ED group and the control group

Individuals with ED exhibited a skewed distribution, encompassing subclinical PE, overt symptoms, and diagnosed psychotic disorders across the full severity spectrum. In contrast, the control group predominantly reported very mild levels of PE; psychotic symptoms were minimally endorsed in the control group, and no psychotic disorders were reported (**Table 2**).

**Table 2:** Frequency and proportion of general lifetime and momentary psychotic experiences (PE), psychotic symptoms, and psychotic disorders (measured with ESM, CAPE, PANSS, and MINI) in an eating disorder group and a control group.

Instrument	Features	Female individuals with an eating disorder, (*N* = 50) *n* (%)	Controls, (*N* = 50) *n* (%)
ESM PE (person mean)	Momentary PE		
=1	Not at all	–	3 (6%)
>1 ≤2	Very mild	11 (22%)	43 (86%)
>2 ≤3	Mild	19 (38%)	3 (6%)
>3 ≤4	Moderate	12 (24%)	1 (2%)
>4 ≤5	Moderate	7 (14%)	–
>5 ≤6	Strong	1 (2%)	–
>6 ≤7	Very strong	–	–
CAPE	Lifetime PE		
Positive frequency scale (at least once item score >1)	Sometimes	48 (96%)	45 (90%)
PANSS	Current psychotic symptoms		
Positive scale (at least once item score ≥4)	Moderate	28 (56%)	1 (2%)
MINI	Psychotic disorder		
	Current	10 (20%)	–
	Lifetime	11 (22%)	1(2%)

The psychosis spectrum phenomena were measured with various instruments. ESM PE (person mean) refers to the average score of the four psychotic experience (PE) items for a specific person across all measurements (beeps). We counted how many individuals (*n*, %) had a person mean PE score equal to 1, between 1 and 2, between 2 and 3, etc. We considered a person mean between 1 and 2 as “very mild”, between 3 and 5 as “moderate”, and between 6 and 7 as “very strong”. The CAPE measures lifetime PE and we calculated how many individuals (*n*, %) had at least once a score >1 (=sometimes) on the positive frequency scale, indicative for PE. The PANSS measures psychotic symptoms and we calculated how many individuals (*n*, %) had at least once a score ≥4 (=moderate) on the positive scale, indicative for clinically relevant. The MINI is a screener for psychotic disorders with a binomial outcome and differentiates in current or lifetime.

The ED group reported significantly higher levels of momentary PE in daily life than the control group. They also reported significantly more lifetime subclinical symptom expression on all CAPE frequency and distress scales (total and positive, negative, and depressive dimension) than controls. Moreover, significantly higher levels of psychotic symptoms on all PANSS scales (total, general, positive, and negative scale) were found in the ED group compared to the control group. Based on the MINI, psychotic disorder was more often present, currently and lifetime, in the ED group than in the control group. The results were adjusted for the *a priori* hypothesized confounders age and education level (**Table 3**).

**Table 3:** Group differences in general psychotic experiences (PE) and symptoms measured with ESM, CAPE, PANSS, and MINI.

Instrument	Female individuals with an eating disorder (*N* = 50)	Control group (*N* = 50)	*B*	95% CI	*p*
ESM momentary general PE, mean (SD)	2.86 (1.33)	1.27 (0.53)	1.57	[1.24, 1.90]	<.001***
CAPE lifetime PE (frequency), mean (SD)					
Positive scale—f	0.30 (0.27)	0.13 (0.10)	0.16	[0.15, 0.17]	<.001***
Negative scale—f	1.20 (0.54)	0.55 (0.36)	0.68	[0.66, 0.70]	<.001***
Depressive scale—f	1.61 (0.62)	0.60 (0.28)	1.03	[1.01, 1.05]	<.001***
Total scale—f	0.85 (0.34)	0.36 (0.18)	0.50	[0.49, 0.51]	<.001***
CAPE lifetime PE (distress), mean (*SD*)					
Positive scale—d	1.46 (0.74)	0.56 (0.53)	0.87	[0.84, 0.90]	<.001***
Negative scale—d	1.57 (0.44)	0.73 (0.42)	0.83	[0.81, 0.85]	<.001***
Depressive scale—d	1.94 (0.53)	0.95 (0.47)	1.00	[0.97, 1.02]	<.001***
Total scale—d	1.65 (0.41)	0.77 (0.39)	0.88	[0.86, 0.90]	<.001***
PANSS current psychotic symptoms, mean (SD)					
Positive scale	11.16 (2.98)	7.54 (1.09)	3.55	[3.44, 3.65]	<.001***
Negative scale	12.06 (4.91)	7.42 (1.16)	4.63	[4.46, 4.80]	<.001***
General scale	26.70 (6.25)	17.64 (2.20)	9.07	[8.85, 9.30]	<.001***
Total scale	49.88 (11.46)	32.60 (3.40)	17.21	[16.81, 17.61]	<.001***
MINI psychotic disorder, *n* (%)					
Current	10 (20%)	0 (0%)	3.23	[1.10, 8.10]-	<.001***
Lifetime	11 (22%)	1 (2%)	2.41	[0.85, 4.70]	.001**

ESM, experience sampling method; CAPE, Community Assessment of Psychic Experiences; PANSS, Positive and Negative Syndrome Scale; MINI, Mini-International Neuropsychiatric Interview. The analyses were corrected for age and educational level.

**p <.01, ***p <.001.

### Association between psychosis spectrum phenomena and daily life factors (NA, self-esteem, and stress) in the ED group

Momentary PE were significantly associated with NA, self-esteem, and stress scores (i.e., activity-related, event-related, and social stress) (**Table 4**). Lifetime positive PE (CAPE positive) were not significantly associated with any of the daily life factors, whereas total lifetime PE (CAPE total frequency and distress) were significantly associated with NA and stress. Total lifetime PE (CAPE total distress) were negatively associated with self-esteem, though insignificant after adjustment for multiple testing (*p* = .055) (**Table 4**). Psychotic symptoms were significantly associated with NA (**Table 4**). None of the daily life factors was significantly associated with psychotic disorder, including self-esteem after adjustment for multiple testing (*p* = .055).

**Table 4:** Associations between 'psychosis spectrum phenomena and other problems in daily life as well as the severity of the eating disorder.

		Momentary PE	CAPE pos. f.	CAPE tot. f.	CAPE pos. d.	CAPE tot. d.	PANSS pos.	PANSS total	MINI current	MINI lifetime
Other problems										
Negative affect	*B*	0.44	0.00	0.12	0.07	0.14	0.61	4.71	0.06	0.16
	95% CI	[0.38, 0.50]	[-0.07, 0.07]	[0.04, 0.19]	[-0.12, 0.26]	[0.05, 0.23]	[-0.10, 1.32]	[2.38, 7.03]	[-0.54, 0.66]	[-0.42, 0.75]
	Adj. *p*	.006**	.922	.012*	.596	.015*	.167	.006**	.911	.681
Self-esteem	*B*	-0.27	-0.00	-0.08	-0.21	-0.14	-0.08	-1.90	-0.78	-0.92
	95% CI	[-0.34, -0.21]	[-0.06, 0.06]	[-0.16, -0.01]	[-0.37, -0.04]	[-0.23, -0.06]	[-0.77, 0.61]	[-4.39, 0.59]	[-1.60, -0.12]	[-1.76, -0.25]
	Adj. *p*	.006**	.969	.175	.174	.055	.852	.173	.108	.055
Activity stress	*B*	0.11	0.08	0.17	0.00	0.19	0.71	2.75	0.67	0.75
	*95%* CI	[0.08, 0.14]	[-0.01, 0.16]	[0.07, 0.27]	[-0.24, 0.25]	[0.07, 0.31]	[-0.24, 1.66]	[-0.75, 6.25]	[-0.18, 1.70]	[-0.06, 1.73]
	Adj. *p*	.006**	.163	.006**	.985	.012*	.231	.208	.231	.167
Event stress	*B*	0.22	0.20	0.31	0.17	0.27	1.32	4.98	0.52	0.45
	95% CI	[0.16, 0.28]	[0.00, 0.41]	[0.06, 0.55]	[-0.42, 0.76]	[-0.04, 0.59]	[-0.99, 3.64]	[-3.55, 13.51]	[-1.39, 2.35]	[-1.42, 2.23]
	Adj. *p*	.006**	.116	.048*	.681	.167	.384	.382	.681	.702
Social stress	*B*	0.16	0.07	0.13	0.04	0.04	0.30	2.50	0.29	0.23
	95% CI	[0.11, 0.21]	[0.00, 0.14]	[0.05, 0.22]	[-0.18, 0.25]	[-0.07, 0.15]	[-0.52, 1.12]	[-0.44, 5.44]	[-0.35, 0.94]	[-0.40, 0.87]
	Adj. *p*	.006**	.116	.010*	.807	.596	.596	.167	.546	.596
Severity ED										
EDE-Q global	*B*	0.43	0.06	0.19	0.21	0.28	0.38	2.69	1.05	1.33
	95% CI Adj. *p*	[0.11, 0.74] .030*	[-0.03, 0.15] .222	[0.09, 0.28] .004**	[-0.04, 0.45] .140	[0.17, 0.39] .004**	[-0.60, 1.37] .438	[-0.89, 6.27] .176	[0.03, 2.34] .131	[0.27, 2.67] .063

Regression estimates of the associations between psychotic spectrum phenomena (measured with ESM, CAPE, PANSS, and MINI) and other problems in daily life as well as the severity of the eating disorder (measured with EDE-Q), corrected for age and education level.

ESM, experience sampling method; PANSS, Positive and Negative Syndrome Scale; CAPE, Community Assessment of Psychic Experiences; MINI, Mini-International Neuropsychiatric Interview; ED, eating disorder; EDE-Q, Eating Disorder Examination Questionnaire.

*p <.05, **p <.01 (significance, with adjusted *p*-values after Benjamini–Hochberg correction for multiple testing).

### Association between the severity of the ED (EDE-Q) and psychosis spectrum phenomena in the ED group

The severity of the ED, measured with the global EDE-Q score, was significantly associated with momentary PE. In addition, no significant associations were found between EDE-Q and positive frequency and positive distress scale of lifetime PE (CAPE positive dimension). EDE-Q was significantly associated with the total scales of lifetime PE (CAPE frequency and distress dimension). EDE-Q was not significantly associated with psychotic symptoms or psychotic disorders.

### Substantiation of using ESM for measuring PE in the ED group

Momentary PE was significantly associated with CAPE total distress and total frequency scale (**Table 5**) in the ED group but was not significantly associated with the CAPE positive dimension. There was also a significant positive association between momentary PE and PANSS positive and total scale symptomatology (**Table 5**).

**Table 5:** Substantiation of using ESM to measure momentary psychotic experiences (PE) for female individuals with an eating disorder.

ESM momentary PE, Mean (SD) = 2.86 (1.33)				
	Mean (SD)	*B*	95% CI	*p*
CAPE lifetime PE				
Positive frequency scale	0.30 (0.27)	0.59	[-0.53, 1.71]	.297
Total frequency scale	0.85 (0.34)	1.03	[0.18, 1.89]	.019*
Positive distress scale	1.46 (0.74)	0.12	[-0.30, 0.54]	.568
Total distress scale	1.65 (0.41)	0.75	[0.06, 1.45]	.035*
PANSS current psychotic symptoms				
Positive scale	11.16 (2.98)	0.11	[0.01, 0.20]	.034*
Total scale	49.88 (11.46)	0.03	[0.01, 0.06]	.014*

Regression estimates of the associations between momentary PE (measured with ESM) and lifetime PE and psychotic symptoms (measured with CAPE and PANSS) in female individuals with an eating disorder, adjusted for age and educational level.

ESM, experience sampling method; CAPE, Community Assessment of Psychic Experiences; PANSS, Positive and Negative Syndrome Scale.

*p <.05.

## Discussion

The present study confirmed the occurrence of the full psychosis spectrum in individuals with EDs (primarily female youth with AN), compared to controls, and—as a next step—examined these phenomena in daily life. The ED group showed significantly higher levels of momentary PE, lifetime PE, psychotic symptoms, and psychotic disorders compared to the control group. Within the ED group, momentary NA, self-esteem, and experienced stress were significantly associated with momentary PE; additionally, NA and stress were significantly associated with lifetime PE. Further along the psychosis spectrum, NA was associated with psychotic symptoms, while none of the daily life factors was associated with psychotic disorder. ED severity (EDE-Q global scale) was associated with momentary and lifetime total PE, but not with psychotic symptoms or psychotic disorders. The significant associations between the momentary PE (ESM), lifetime PE (CAPE), and psychotic symptoms (PANSS) indicate weak convergent validity for assessing PE with ESM in individuals with EDs.

### Occurrence of psychosis spectrum phenomena in the control group and ED group

In the control group, the proportion of individuals reporting *very mild* lifetime and momentary PE was very high (90% and 94%, respectively). These rates substantially exceed those reported by van Os et al. (9) for the general population (8%–33%). This discrepancy likely reflects differences in measurement: the review focused on PE just below the clinical threshold, whereas our study captured more subtle forms of PE. The high percentages observed here are consistent evidence that these *very mild* PE are common in the general population (46). Using ESM, we differentiated between intensity levels of momentary PE and found that only 8% of controls reported PE rated as at least *mild* or higher, a figure that aligns with the previous population-level estimates.

In the ED group (predominantly individuals with AN), the proportions reporting *very mild* lifetime and momentary PE were also very high (96% and 100%), but only slightly higher than in our control group. ESM data further indicated that 78% of the individuals with ED showed mean PE levels of at least mild intensity in daily life. To our knowledge, this is the first study to use ESM to detect subclinical PE levels in an ED sample. Assessing these subtle experiences is important, as they can be distressing and may represent early stages of more severe psychotic development (17, 47). While most previous research focused on psychotic disorders, symptoms, or at-risk mental states (6), examining these early micro-phenotypes may support the development of strategies for early detection and prevention of more severe psychopathology.

In the present control group, 2% of female youth reported psychotic symptoms and 0% screened positive for psychotic disorders (acute psychosis was an exclusion criterion). These rates are slightly lower than those reported in a general population sample (9), where 4%–17% experienced psychotic symptoms and 1.7% met the criteria for a psychotic disorder. This difference may reflect our recruitment strategy: by excluding individuals currently receiving psychological treatment, we may have selected a control group with lower psychosis vulnerability.

In the present ED group, the proportion of individuals experiencing psychotic symptoms (56%) or a psychotic disorder (current: 20% and lifetime: 22%) was substantially higher compared to the control group. This prevalence of psychotic symptoms is consistent with studies in comparable outpatient samples of female youth with AN and BN, which report rates ranging from 21.7% to 84.3% (48–50). When focusing specifically on psychotic symptoms measured with the same instrument (PANSS), the PANSS total score in our study (*M* = 49.88, SD = 11.46) was comparable to other reports of outpatients with AN (*M* = 62.78, SD = 8.34) and BN (*M* = 47.90, SD = 5.84) (51) as well as a small outpatient sample of female ED youth (*M* = 43.2–56.5, SD = 7.2-0.7) (52).

Although the 20% prevalence of psychotic disorders in our study appears high relative to the 8% reported in the meta-analysis of Drymonitou et al. (6), it aligns with several individual studies included in that review—for example, 13% (53), 20% (52), and 52% (54)—all conducted in intensive ED treatment settings (inpatient or second-line mental health care) similar to the present study. A recent study on newly admitted outpatients aged 16–35 from a second-line mental health care (*N* = 736), not included in this meta-analysis, reported a comparable prevalence of psychotic disorders (18.7%) among individuals screening positive for an ED (55).

These results support the assumption that all levels of the psychosis spectrum are present in an ED group of young women, primarily with AN, and subclinical psychosis levels were comparable to those in the control group. However, toward the more severe end of the spectrum, the difference between the ED group and “healthy” controls became pronounced, with individuals with ED showing markedly higher levels of (clinical) psychosis vulnerability.

### Wider symptom dynamic of psychosis spectrum phenomena in the context of daily life in the ED group

All contextualized factors (NA, self-esteem, and three types of stress) were significantly associated with higher levels of momentary PE. In addition, NA and stress were associated with lifetime PE (CAPE total scales). Although the direction of these associations cannot be determined from our data, it is plausible that these factors contribute to the emergence of PE in the ED group and may therefore constitute underlying mechanisms. Existing research supports this interpretation: evidence indicates that low self-esteem and heightened sensitivity to daily events increase stress, which subsequently predicts NA; stress and NA together are established predictors of PE (24, 56, 57). Because these contextualized factors are also considered core mechanisms driving ED symptoms (2, 58, 59), our findings are consistent with the hypothesis of shared mechanisms underlying EDs and vulnerability to psychosis (8, 23, 48).

We hypothesized that the contextualized factors would also be associated with the CAPE positive dimension. However, after correcting for multiple testing, no significant associations were found between daily life factors and the CAPE positive dimension. This suggests that ESM may be more sensitive than the CAPE in detecting such associations, as it captures immediate, context-dependent experiences, whereas the CAPE represents a more reflective, long-term experience. Indeed momentary PE levels were higher in the contexts of increased NA, lower self-esteem, and higher stress. Alternatively, lifetime negative and depressive symptoms may be more strongly linked to current NA, stress, and low self-esteem than to lifetime positive PE, which could explain why significant associations were observed for the CAPE total score but not the CAPE positive score.

Regarding psychotic symptoms and psychotic disorders, NA was the only contextualized factor significantly associated with psychotic symptoms. Methodologically, this may reflect a sample too small to detect associations between contextualized factors (NA, self-esteem, and stress) and psychotic symptoms or disorders using cross-sectional questionnaires (PANSS and MINI). One might question whether these associations are driven by comorbid conditions, such as mood disorders, rather than by psychotic features per se. However, the high degree of multimorbidity in individuals with EDs underscores their complexity, which also influences psychosis spectrum phenomena; psychosis in mental conditions is generally considered an indicator of severity (17, 47). Thus, NA, self-esteem, and stress appear to be transdiagnostic factors contributing both to psychosis and other comorbid psychopathology.

### Association between psychosis spectrum phenomena and ED severity

Partly consistent with our hypothesis of a positive association between ED severity and the psychosis continuum, the findings of the present study showed that higher EDE-Q global scores were associated with increased levels of both momentary and lifetime PE. In contrast, ED severity was not related to psychotic symptoms or to the presence of psychotic disorders, suggesting that the association may be limited to subclinical manifestations of psychosis. If this is not due to lack of power, a tentative explanation could be that in young women with a relatively short duration of the ED (as in the current study sample), PE do not necessarily progress to a full psychotic disorder but may instead reflect the development or persistence of another macro-phenotype, such as ED (11, 60). Future research involving a more severe and older ED sample may help clarify this issue.

### Clinical implications

Given that psychotic spectrum phenomena were relatively common in this population of young women with EDs (mostly AN) and considering that mild PE may evolve into psychotic symptoms or even a psychotic disorder, prevention and intervention strategies for psychosis risk states may be warranted and deserve greater attention in ED diagnostics and treatment. Such strategies could include psychotherapeutic interventions and, when appropriate, antipsychotic medication. The latter is only recommended in the case where psychotic symptoms or psychotic disorders are present, as subclinical psychosis is not treated with antipsychotic medication according to current guidelines (61).

Furthermore, the use of ESM may help identify and monitor PE in daily life among individuals with EDs, signaling when early interventions are needed. In the current study, self-esteem, NA, and stress were associated with psychosis spectrum phenomena. Because these psychological and contextual factors also represent shared underlying mechanisms of EDs (2), it may be crucial to address them in ED treatment to prevent self-sustaining, decontextualized symptom patterns and to improve recovery rates.

Based on future replications on transdiagnostic mechanisms of psychosis micro-phenotypes in EDs, standard ED treatments could be complemented with interventions targeting emotion and stress regulation, self-image, and maladaptive schemas—such as schema-focused therapy or EMDR addressing low or damaged self-esteem—to reduce relapse risk. This population, which typically exhibits severe deficits in emotion regulation and self-worth, may benefit more from additional treatments in parallel to standard ED treatment (aiming recovery of physical health, normal eating habits, and ED symptoms). Early access to these interventions appears crucial for prevention and for interrupting pathological development in youth. Future research should explore whether symptom patterns might vary in older individuals and in populations with long-standing EDs compared to female youth in order to clarify the potential differential clinical implications for these populations.

### Strengths and limitations

One of the strengths of this study is that it answers the call for interdisciplinary research (5): by quantitatively examining underlying factors of psychotic spectrum phenomena in individuals with EDs in their daily lives using ESM. By measuring PE in the natural context of daily life, alongside widely used instruments mentioned in the psychosis guideline (CAPE and PANSS), the study was able to capture a wide range of liability along the psychosis continuum. Including contextualized factors and broader symptom dimensions may help expand the focus of diagnostics and treatment within this target group.

Another strength is the narrow age range of the sample (15–25 years), which aligns with the typical early age of onset for EDs and psychosis spectrum phenomena (32). This reduces the influence of secondary (treatment) effects that may result from long-term illness trajectories.

A limitation of the study is that participants were recruited from a single ED center, which restricts generalizability. In addition, only individuals from an outpatient setting were included, further limiting the extent to which the findings can be generalized. Furthermore, we did not collect information about whether or not the participants used psychotropic medication, implying that we may be underestimating the occurrence of (subclinical) psychosis, NA, or stress in this target group. Another limitation is that while the sample size was adequate to analyze the ESM data (i.e., 100 ESM observations per person), it was relatively small for the retrospective questionnaires and interviews.

## Conclusion

This study shows that psychosis spectrum phenomena—from subclinical PE to psychotic disorders, assessed as current, lifetime, and in daily life—are highly prevalent in female youth with EDs, predominantly AN, at the start of treatment. This underscores the vulnerability of this group and their need for comprehensive care. These (subclinical) psychosis spectrum phenomena were associated with momentary experience of NA, stress, and/or low self-esteem, all of which are known transdiagnostic factors in EDs and may point to shared underlying mechanisms.

Moreover, the study found evidence that subclinical PE were associated with ED severity. This suggests that ESM can be used exploratively to investigate PE and their underlying factors in daily life among individuals with EDs, thereby informing early interventions or relapse prevention strategies. Clinical interventions focused on transdiagnostic factors (NA, stress, low self-esteem), as well as clinical staging approaches from psychosis applied to ED diagnostics and treatment, may help strengthen resilience in individuals with EDs.

## Data Availability

The raw data supporting the conclusions of this article will be made available by the authors, without undue reservation.
